# A qualitative analysis of the social and cultural contexts that shape screen time use in Latino families living on the U.S.-Mexico border

**DOI:** 10.1080/17482631.2020.1735766

**Published:** 2020-03-02

**Authors:** Cristina S. Barroso, Andrew E. Springer, Christopher M. Ledingham, Steven H. Kelder

**Affiliations:** a Department of Public Health, University of Tennessee, Knoxville, TN, USA; b Michael & Susan Dell Center for Healthy Living, Department of Health Promotion and Behavioral Sciences, School of Public Health, University of Texas Health, Austin, TX, USA; c Health and Human Performance, The University of Texas Rio Grande Valley, Brownsville, TX, USA; d Michael & Susan Dell Center for Healthy Living, Department of Epidemiology, Human Genetics, and Environmental Sciences, School of Public Health, University of Texas Health, Austin, TX, USA

**Keywords:** Screen time, Latino immigrant families, social contexts, cultural contexts, *familismo*, *respeto*

## Abstract

**Purpose**: The purpose of this study was to understand how first generation Latino parents, whose primary language is Spanish and live in a *colonia* on the U.S.-Mexico border, use screen time in their homes.

**Methods**: A purposeful sampling approach was used to recruit eligible parents of pre-adolescents (ages 9–14) who were native Spanish speakers, and living on the U.S.-Mexico border. Three focus groups in Spanish (two with mothers and one with fathers) were conducted. Data were codified using a general inductive approach based on grounded theory. A consensus process was repeated until a final codebook was developed.

**Results**: Screen time allowed parents to foster *familismo* (family cohesiveness and bonding) and *respeto* (respect). Parents knew that a healthy balance of media use is important, but broader social contexts (marital discord and economics) challenged the enforcement of familial screen time rules and parents were often permissive.

**Conclusions**: Our study addressed research gaps by examining the understudied social and cultural contexts (practices, routines, rules, and beliefs) that shape children’s screen time use among a sample of Latino immigrants living on the U.S.-Mexico border. This sample of parents indicated that *familismo* and *respeto* (i.e., cohesiveness and bonding) influence familial decision-making including screen time.

## Introduction

Screen time is the amount of time a person spends using a device with a screen such as a television (TV), computer, video game console, mobile phone, or tablet. Excessive screen time and other sedentary behaviours are associated with an increased risk for obesity, lower physical fitness, greater anti-social behaviours, and lower academic achievement in children and adolescents (Hancox, Milne, & Poulton, 2004; Robinson et al., 2017; Tremblay et al., 2011). Although the displacement of physical activity with screen time in children is not strongly associated with obesity (Ramsey Buchanan et al., 2016), the lack of physical activity is associated with weight gain and an increased risk of developing type 2 diabetes (T2D), cardiovascular disease, and some forms of cancer (2018Physical Activity Guidelines Advisory Committee, 2018). Screen time is also associated with the consumption of high caloric, low nutrient foods in both children and adults (Ford, Ward, & White, 2012; Pearson & Biddle, 2011). Finally, incessant screen time and exposure to blue light, particularly around bedtime, are related to sleep disturbances (Bruni et al., 2015). Hence, too much screen time may usurp healthy behaviours and can be detrimental to children’s health.

While some evidence indicates that traditional TV watching among United States (U.S.) adolescents has decreased (Gingold, Simon, & Schoendorf, 2014; Pew Research Center, 2018; Ryu, Kim, Kang, Pedisic, & Loprinzi, 2019), findings from these same studies also suggests that overall screen time has increased because adolescents use new and different platforms (i.e., computers, cell phones, and other mobile devices) to primarily access social media (i.e., Snapchat, YouTube, and Instagram) (Gingold et al., 2014; Pew Research Center, 2018; Ryu et al., 2019). Data from the 2003 to 2015 Youth Risk Behaviour Surveillance System, a nationally representative sample of 9–12^th^ grade students (typically 14–19 years old) in the U.S., showed that TV watching decreased from 4.1 hours per day (h/1day) to 3.3 h/day, while computer use increased from 3.2 h/day to 4.0 h/day (Ryu et al., 2019). Similar screen time trends have been observed in children and adolescents from Australia (ages 0–12) and Europe (ages 11, 13, and 15) (Bucksch et al., 2016; Tooth, Moss, Hockey, & Mishra, 2019). Furthermore, children from low-income, low parent education families spend more screen time per day than children from high-income, higher parent education families (Rideout, 2017).

Most of what is known about screen time behaviours and family screen time rules in the U.S. is based on middle class white children and their families (American Academy of Pediatrics Council on Communications and Media, 2016; Taveras, Hohman, Price, Gortmaker, & Sonneville, 2009); groups not disproportionately affected by health disparities. Ethnic minority adolescents in the U.S. have higher TV watching percentages than their white counterparts (Gingold et al., 2014; Taveras et al., 2009). Some research has found that 67% of minority parents (i.e., Latino and African American) allow their children aged 2–13 years to have a TV in their bedroom (Taveras et al., 2009). Latino children have lower levels of moderate to vigorous physical activity (American Academy of Pediatrics Council on Communications and Media, 2016; Andersen, Crespo, Bartlett, Cheskin, & Pratt, 1998; Gordon-Larsen, McMurray, & Popkin, 2000) and are more likely to be inactive (Dugas et al., 2008) than their white counterparts Hence, more research is needed to explore how the home environment may influence sedentary behaviours. Although it is known that Latino parents are concerned about children’s screen time (Gingold et al., 2014), little is known about the social cultural contexts of screen time in Latino children, in particular, low-income immigrant Latino families from the U.S.-Mexico border living in *colonias*. A *colonia*, Spanish for neighbourhood, is an unannexed section of a U.S.-Mexico border city/town lacking basic services such as paved roads and waste management. *Colonia* residents have higher poverty rates, lower educational attainment, and greater prevalence of chronic health conditions such as obesity and T2D than the U.S.as a whole (De La Puente & Stemper, 2003).

Given the lack of investigation into familial screen time practices among first generation Latino families, we seek to explore the social and cultural contexts, specifically practices, routines, rules, and beliefs, that shape how screen time is used. The purpose of this exploratory qualitative study was to understand how Latino immigrant parents, whose primary language is Spanish and live on the U.S.-Mexico border in a *colonia*, engage with screen time in their homes.

## Materials and methods

### Study design and sample

The internal review board of a large research institution approved all research protocols and materials (IRB number: HSC-SPH-07-0619). We used purposeful sampling for this exploratory focus group study. Focus groups, as a qualitative research tool, are a cost-effective method to obtain and understand perceptions, opinions, feelings, and behaviours about a particular phenomenon in a group setting, where group members have similar characteristics. Eligible focus group participants were parents of pre-adolescents (ages 9–14), native Spanish speakers, and living on the U.S.-Mexico border in a *colonia*. Parents of pre-adolescents were selected because screen time habits of pre-adolescents are diverse (use of more than one platform) and still influenced by the home environment. Furthermore, at the time of this study, a community-wide media campaign (Reininger et al., 2015) to increase physical activity and fruit and vegetable consumption in adults was being implemented throughout the area, including the selected *colonia*. Similar efforts with pre-adolescents were being planned, but how Latino families engage with screen time in their homes needed to be investigated first. This specific *colonia* is among the poorest areas of the U.S. (the per capita income is 8,518 USD) and less than 50% of adults living in the *colonia* have a high school education (U.S. Census Bureau).

A *promotora* (community health worker) trained in research procedures recruited eligible parents living in this specific *colonia* on the U.S.-Mexico border via word of mouth. Word of mouth was used to recruit participants because the trained *promotora* had an established relationship with *colonia* residents as part of the community-wide campaign. The day before each scheduled focus group session, the *promotora* called eligible parents to confirm their attendance. All of the recruited female participants attended the focus groups, but six of the 10 male participants recruited for the study did not attend. Participants also completed a brief socio-demographic survey to describe their characteristics: age, gender, number of adults and children in the household, and information on screen time habits and services available in the home such as the types of screens, TV in the bedroom, and Internet, cable, and satellite.

### Focus group guide & data collection

We developed a semi-structured focus group guide based on a literature review related to screen time family policies in the home (2018Physical Activity Guidelines Advisory Committee, 2018; Hancox et al., 2004; Taveras et al., 2009). English/Spanish bilingual study staff translated and back-translated the questions to ascertain accuracy. The questions were then pilot tested with other bilingual university *promotoras*, not associated with the current study. Their feedback resulted in a 10-item, with probes, open-ended semi-structured focus group guide (Table I). Focus group moderators were also trained to use probes, such as “tell me more about … ” to obtain detailed and meaningful responses from the participants about topics not included on the focus group guide.

**Table I.** Semi-structured focus group guide: 10 open-ended questions and probes

Item
1. Tell me about television (TV) use in your home or with your family. When should parents worry about their children watching too much television?
2. Tell me about the good things of having a TV in your child’s bedroom. Is there anything that worries you about having a TV in your child’s bedroom? What do you think would happen if you took out the TV from your child’s bedroom?
3. What other screens (e.g., video games, computer, handhelds, DVD, TiVo) do your children spend time with? Tell me your concerns about these.
4. If any, what are the family rules or guidelines for TV use? Tell me about using TV as a reward or punishment.
5. What would happen if you set limits on TV or video game playing? What would be the child’s reaction? What would be the family’s reaction? What would you do if there were discipline issues?
6. What are the things that you or your family eat and drink while watching TV? When should parents worry about the amount of food or the quality of the snacks that their kids are eating while watching TV?
7. Describe what healthful snacks are.
8. Tell me about you and your family’s everyday life.
9. Tell me about your family’s activities. (The ones that you and your family like to do.) Why do you think your family enjoys doing these activities?
10. How have TV commercials affected you or your family? Tell me what happened after you or your child saw the TV commercials?

A total of 20 parents participated in the study: two focus groups with mothers and one with fathers. The mother focus groups consisted of eight women each and occurred during a weekday morning at a church. The fathers met during a weekday evening at a community centre. These locations, a church and a community centre, were selected because they were convenient and easily accessible by all participants. Participation in focus groups was voluntary, anonymous, and confidential. All participants provided written informed consent for participation. Participants received 10 USD gift cards from a national retailer and nutrient-dense refreshments for their participation in the study.

Focus groups were systematic and sequential as recommended by Krueger and Casey (Krueger & Casey, 2014). Research staff trained in focus group methodology conducted all three focus groups in Spanish. Focus groups were conducted over a 4-month period. The same Mexico-born female moderator conducted the two female focus groups and a Mexico-born male moderator conducted the male focus group. Additionally, as endorsed by Krueger and Casey (Krueger & Casey, 2014), three Spanish speaking female observers took notes at each focus group. Each focus group started with a general overview of the study, the focus group process, and the anonymity and confidential nature of the study. Research staff obtained informed consent from all participants before the start of each focus group. On average, the focus groups lasted 90 minutes. All focus groups were digitally audio-recorded. Research staff members transcribed the digital recordings verbatim and translated the transcripts into English. For quality control, the lead author crosschecked both the Spanish transcripts and the English translated transcripts for accuracy. We present the data from the English language translations of the transcripts here.

### Analysis

We used IBM SPSS Statistics version 20.0 to calculate frequencies, means, and standard deviations (SDs) for the socio-demographic data. A thematic analysis approach based on grounded theory was used to analyse the focus group data, which allows themes to emerge from the data (Strauss & Corbin, 2007). All coding was iterative and collaborative. To establish codes, categories, and sub-categories, two of the investigators manually (paper and pen) and independently reviewed, abstracted, and coded a segment (four pages) of the de-identified transcript from the first focus group with mothers. The researchers then met to discuss their codes, categories, subcategories, and rationale. After the initial meeting, the two investigators continued to manually and independently review, abstract, and code an additional four pages. The two researchers independently developed provisional codebooks (code name and description, categories, subcategories, and examples for each). Then the researchers met to discuss their codes (agreements and discrepancies of assigned codes) and merged their provisional codebooks. This consensus meeting process was repeated four more times until the entire transcript was coded. These codification and abstraction processes were done iteratively to test, revise, and refine the thematic classifications and to develop a final codebook. Themes identified were further compared and contrasted for similarity and/or difference in responses by a third investigator.

At each consensus meeting, to assess coding agreement, we calculated the percentage of agreement for the most frequently coded sections of the transcript. Coding agreement, inter-coder reliability, was when both coders assigned the same code to the main idea of a segment from a transcript (Burla et al., 2008). The overall inter-coder reliability for the transcript was 91.4%. Using the final codebook, we used NVivo to code the other two transcripts.

Consensus between the three investigators regarding codification and abstraction resulted in five identified themes related to the socio-cultural environment of the participants. Categories and themes are presented and appropriate quotes were selected (Strauss & Corbin, 2007).

## Results

### Socio-demographic characteristics and screen time habits

Table II depicts the socio-demographic characteristics and screen time habits of the sample. Parents in the purposeful sample were born in Mexico. The average age of mothers was 36 years old (SD = 5.2; n = 16) and the average age of the fathers was 34.3 years old (SD = 5.1; n = 4). The majority of parents (88.9%) were concerned about the amount of TV watched by their children, 63.2% reported to have screen time rules in their homes, and 45% stated that it was difficult to establish screen time rules. Most households had a TV in the child’s bedroom (80%) and provided a multiple-screen environment; all households had cable/satellite TV and/or Internet services. Although the semi-structured focus group guide allowed for probing questions, most of the probes primarily focused on TV and televised programming.

**Table II.** Socio-demographic characteristics and screen time habits of participants (N = 20)

Characteristics/Habit	Percent
Female	80%
Age	Mean = 35.7; SD* = 5.1
Born in Mexico	100%
Adults in the home	Mean = 2.2; SD = 0.8
1	17.6
2	47.1
3	29.4
4	5.9
Children in the home	Mean = 2.2; SD = 0.8
1	15.8
2	52.6
3	26.3
4	5.3
High school diploma/GED	57.9
Concerned about amount of TV watching	88.9
Screens in the home	
Computer	50.0
Video games	50.0
DVD/VCR	75.0
TiVo	5.0
Cellular phone	70.0
Digital media player	15.0
Media service	
Cable/satellite TV	77.8
Internet	25.0
TV in child’s bedroom	80.0
Difficult to establish screen time rules	45.0
Screen time rules in the home	63.2
TV as a reward	35.0
TV as a punishment	85.0

*SD: standard deviation.

### Identified themes

We identified five key themes: (1) use of screen time in the home; (2) screen time rules, (3) screen content, (4) screens and food, and (5) alternate activities. Table III shows the categories within each identified theme by focus group.

**Table III.** Categories within each identified theme by focus group

Mothers Group One (n = 8)	Mothers Group Two (n = 8)	Fathers (n = 4)
**Use of Screen Time in the Home**		
Bad habit (1) Hooked (2) Not a problem (2) Some children prefer video games/cell phones over TV (2) To teach (2) Babysitter (2) Placate—not struggle with fussy kids (3)	Bad habit (2) Placate—not struggle with fussy kids (2) TV in the bedroom (2) Babysitter (2) Hooked (3) Some children prefer video games/cell phones over TV (3) To teach (5)	Placate—not struggle with fussy kids (2) To teach (2) Inexpensive entertainment (3)
**Screen Time Rules**		
Teach kids what our parents taught us (2) Husband needs to follow what I suggest (3) Some kids pay attention others don’t (3) Take it away—no screens (3) Rules for use (11)	Husband needs to follow what I suggest (2) Take it away—no screens (2) Teach kids what our parents taught us (3) Rules for use (6)	Teach kids what our parents taught us (2) Some kids pay attention others don’t (2) Take it away—no screens (3) Desidia (ambivalence) (5) Rules for use (7)
**Screen Content**		
Pines for childhood shows (3) American TV shows bad influence (4) Commercials—adult content (4) Internet/video games—adult content (5)	Channel surfing—adult content (1) Internet/video games—adult content (3) Commercials—adult content (4) Pines for childhood shows (4) American TV shows bad influence (5)	American TV shows bad influence (1) TV content confuses children (1) Telenovelas—nudity (2) Liberal indoctrination (3) Internet/video games—adult content (4) Pines for childhood shows (4) Commercials—adult content (5)
**Screens and Food**		
Diabetes a problem, but I give McDonald’s (1) Other people’s kids eat junk food (3) Food and health (4) Hot Cheetos, but snacking not a problem (4) TV–induced food cravings (6)	Modified diet for health (1) Fruit when watching TV (1) Other people’s kids eat junk food (2) TV–induced food cravings (2) Hot Cheetos, but snacking not a problem (3) Food and health (3)	TV and eating don’t mix (2)
**Alternate Activities**		
Not discussed	Screen time and obesity (1) Outside they are happy (2)	Movie theatre (1) Family gatherings (2) Reward kids with family fun (2) Wife doesn’t have time (3) Outside they are happy (4)

Frequencies are shown in parentheses.

### Use of screen time in the home

All of the parents discussed the use of screen time in the home. All families had more than one TV set. Children’s screen time included use of TV, computers, cell phones, and video games (both hand-held and those connected to a monitor), whereas parental screen time primarily was TV. Some parents reported that excessive screen time was not an issue, while others described children as “hooked” to screens and brainwashed by TV. Mothers reported constant use of TV even if no one was watching. Mothers did not perceive their constant TV use as a screen time role-modelling behaviour for their children. Children viewed both U.S. (e.g., ABC, CBS, NBC, cable networks, Univision, and Telemundo) and Mexico (e.g., Televisa and TV Azteca) TV programming. Screens served as a diversion to amuse children when other options were unavailable, as a babysitter, as a mediator to avert conflicts, arguments, and disturbances, and as an educational tool.

Mother Focus Group 2 (FG2): *I’m hooked, whatever I have to do it’s [the TV] on. I might not be watching but I’m listening. Sometimes my husband turns it off. He tells me, “You’re not even watching.” And then, the kids … when they go to their bedroom, they also have the TV on.*

Father: *Well, it’s important because when you don’t work, I mean you don’t have the money to go out, then that’s all there is. You rent a movie and you watch it. I believe it’s essential, but with limits.*

Father:*Not to struggle [with the kids] and for them [the kids] not to be fussy or annoying others.*

Mother Focus Group 1 (FG1): *I like “La Rosa de Guadalupe” [famous Mexican drama with a moral storyline]. I make them watch it with me so they can learn, by the end they are crying. This show teaches them something.*

### Screen time rules

Screen time rules addressed use, punishments, and compliance with rules. All parents discussed the importance of screen time rules in the home, in particular, how schoolwork supersedes use of screens. Parents emphasized that they are trying to pass down the lessons and philosophy of their parents, especially since children are influenced by peers. All families limited screen time as punishment; however, enforcement was a challenge. Compliance with household rules was dependent on the individual child because some children obey and others do not. Fathers discussed how parents did not consistently enforce their own rules because of *desidia* (ambivalence). The mothers mentioned that for children to comply with family rules, husbands (fathers) must support their judgements and decisions. Only one mother stated that her family established a screen time rule for weight management.

Mother FG1: *They know that there are rules. My son’s grades were dropping and my husband banned TV.*

Father: *One is also to blame because you tell them, “You are not allowed to watch TV, you are punished.” Then you say, “If you behave you can watch TV.” The punishment is lifted.*

Father: *It is desidia [Spanish for “ambivalence”], because if one says, “No, they don’t listen to me.” It’s carelessness, indolence because kids do listen, but one doesn’t want to be bothered with prohibiting things because if you tell them don’t do this, don’t do that, they talk back and [one doesn’t want] to make the child angry or whatever.*

Mother FG1: *Especially in front of the kids … when your husband doesn’t back you up since he isn’t home and doesn’t know what happened and then he lets the kids watch something or lets the kids chat on the computer.*

### Screen content

All parents noted the mature/adult nature (sex, drugs, narco-trafficking, and violence) of TV programming, advertisements, video games, and on the Internet. Parents yearned for programming from their youth (in particular, “El Chavo del Ocho” and “Viruta y Capulina”) to U.S. children’s shows. “El Chavo del Ocho” is a Mexican satirical sitcom, originally broadcasted 1972 to 1980, that centred on the trials and tribulations of a poor orphan boy. “Viruta y Capulina,” is a Mexican comedy duo, whose heyday was 1956 to 1966, featured in films, radio, theatre, and comic books. Parents stated that American programming and fictional U.S. English-speaking TV characters were negative influencers. Fathers believed that screens indoctrinated modern and liberal attitudes in the family and in society.

Mother FG1: *My nephew’s grades dropped because he has an X-Box and the games are violent. Now, my son wants one. He begs me for one. My husband asks if he should buy him one. I told him, “No!”*

Father: *Back in the day when they aired cartoons, just cartoons and movies of “Capulina and Viruta.” It was wholesome, nothing violent, and, now the women in telenovelas are in their bathing suits and they bend over. Not telenovelas, not cartoons, nothing is safe, you need to be vigilant.*

Mother FG2: *The kids on TV shows are rebellious. They answer back, they do things they shouldn’t, they are out of control. Recently my daughter started to tell me that she does not want to live with me anymore. Until one day I heard the TV … if I call to her, she ignores me, or if I speak sternly, she raises her voice to me. She watches that show and she sees this girl doing whatever she wants with her dad; she leaves without permission, her dad is always after her, she shouts louder than her father and leaves him talking to himself. My daughter was becoming like that.*

Father: *Because things weren’t shown like they are now. The father was stricter, but now … That is why what’s happening is happening … Before they followed the fathers’ rules.*

### Screens and food

There was a disconnect between what mothers said their families ate, especially during screen time, and what they believed. Mothers commented on the influence of food advertisements and food product placement in TV shows on their children’s eating habits as well as their own, but most stated that snacking on calorie-dense foods and consumption of poor diet quality foods, in general, were not a problem for their families. Mothers believed that other people’s children had poor eating habits, not their own. Although mothers noted the role of food habits on health, including poor food choices, overeating, and childhood obesity, only one mother mentioned that she made changes to the family’s diet because of health concerns. None of the fathers discussed food habits and its influence on health. Two fathers discussed the restriction of foods to dining areas during screen time to maintain cleanliness.

Mother FG1: *They want Dairy Queen instead of craving what we have in the kitchen.*

Mother FG2: *I let my kids eat all the fruit they want when watching TV, but I limit Coke. My children will have half a Coca-Cola. Maybe once a week we have pizza.*

Mother FG1: *I have a niece, she’s 8, very chubby, she’s always eating. She lives in Dallas, she visits me … they are coming for Christmas. I tell her we have rule: if you want a snack you can only have fruit. She loves a big bag of hot Cheetos, but I won’t let her snack. My sister’s house is full of frozen foods, I cook … so now my nieces want my sister to cook like me. Everything is frozen, they eat that and they are very chubby.*

Father: *I don’t like it because when they are served food they go to bed to watch TV. One thing that truly upsets me more than anything is that they must learn that the table is for eating, the bed is for rest, and the TV is for watching. And besides eating there [in bed], they make a mess.*

### Alternate activities

Fathers favoured activities that combined family unity and physical activity as alternatives to screen time. They mentioned picnics, watching movies at theatres, visiting family members across the border, walks, soccer, volleyball, bicycle rides, and swimming. Two fathers remarked that marital discord minimized participation in physical activity as a family. Only two mothers talked about physical activity as an alternate to screen time, but only about their children’s physical activity. One mother connected screen time (sedentary behaviour) with childhood obesity; however, she did not attribute her child’s overweight status to screen time.

Father:*For me, soccer … my son plays soccer so does my daughter. All of my family likes soccer.*

Father: *I believe going to the park, riding a bike, go for a walk, but it’s a two-way street. I would need to speak with my wife because sometimes she says that she doesn’t have time, that she’s tired, that she was doing housework all day, but above all the couple should be in agreement.*

Father: *Because even though one gets home tired, is annoyed more than anything else because of marital problems, you fight, and the ones who suffer are the kids.*

Mother FG2: *If I take them outside they are happy. They could spend the entire afternoon playing outside: riding their bikes, on the skateboard, running, playing ball, they play whatever.*

## Discussion

This exploratory focus group study examined and described the social and cultural contexts (i.e., practices, routines, rules, and beliefs) of screen time in the home of Latino families living on the U.S.-Mexico border. The themes that emerged from this research provide a foundation for further confirmatory research and potential intervention strategies to reduce screen time and other sedentary behaviours.

Similar to previous studies, our findings show that for Latino families living on the U.S.-Mexico border, screen time served as a multi-purpose parenting tool with functions that included babysitting, entertaining, education, and sustaining family harmony (He, Piché, Beynon, & Harris, 2010; Taveras et al., 2009). Two traditional Latino values, which influence parenting style, are *familismo* (familism: the orientation, obligation, and accountability to the family) and *respeto* (respect: both respect and obedience to parents and other authoritative family members) (Calzada, 2010). Screen time helped parents to foster *familismo*: address household responsibilities, prevent disputes, educate, and amuse children. However, screen time was also a menace to *respeto* primarily because of more mature content (compared to when parents were children). Parents believed that screen time taught children to disrespect parental authority and encouraged disobedience. This dissonance that parents experience is not addressed by current screen time interventions (Sanders, Parent, Forehand, Sullivan, & Jones, 2016). Parents knew that a healthy balance of media use is important, but broader social contexts (marital discord and economics) challenged the enforcement of familial screen time rules and rules were often permissive. Fathers used the term *desidia* to describe the reason for leniency. Translation of *desidia* has many nuances; including indecisiveness, ambivalence, uncertainty, doubt, hesitation, inaction, negligence, indolence, carelessness, procrastination, and laziness. Future research could focus on discerning which translation of *desidia* describes the parental experience and how to counter *desidia*. Furthermore, screen time reduction strategies should reinforce the values of *familismo* and *respeto*. For example, future interventions could help families to identify parent-approved screen content that have cultural significance (e.g., “El Chavo del Ocho”) (American Academy of Pediatrics Council on Communications and Media, 2016; Robinson et al., 2017).

In this sample, only mothers discussed the influence of food advertisements and food product placement on TV and familial snacking during screen time. Mothers were responsible for food and snack offerings and most claimed to offer healthful options (ready-to-eat fruits and vegetables). The mothers linked consumption of foods of poor nutritional value with obesity and other health outcomes, yet most believed this to be a common problem in other families but not their own. Although skills-based interventions reinforce the health reasons for restricting snacking during screen time, research on parental perceptions of the home food environment and strategies to help parents link the home food environment and familial dietary behaviours to obesity are warranted (McGuire, Hannan, Neumark-Sztainer, Cossrow, & Story, 2002; Taverno Ross et al., 2018; Thompson et al., 2018).

Fathers deemed physical activity as a viable substitute for screen time, but suggested that minimal engagement in familial physical activity occurred because their wives do not or will not engage in physical activity. It is important to note that the mothers did not equate household tasks with physical activity and that the child-centred questions may have caused the mothers to focus only on their children’s physical activity. Future research should explore the barriers and facilitators for familial leisure-time physical activity. Interventions should help families to engage in fun and easy active play games, increase both self- and collective efficacy in physical activity, and augment the level of familial social support (praise for and from all family members) for daily physical activity (Larsen, Noble, Murray, & Marcus, 2015; Taverno Ross et al., 2018).

## Limitations

Although our study uncovered significant insights of the social and cultural contexts that shape screen time use, it has several important limitations. As an exploratory qualitative study, the purpose was to understand the context of the experiences and perspectives of the parents. These findings are not generalizable to other populations, but results may be transferable to similar low-income Latino immigrant families in the U.S. Additionally, the Latino immigrant families in this study were from Mexico and are not representative of all Latino immigrant families in the U.S. Nonetheless, the notes from three observers at each focus group session were used to supplement the transcriptions and the English translations. This allowed for examination of the accuracy of the data collected, which helped to establish the credibility of the data, and ultimately, the trustworthiness of the study (Shenton, 2004). In particular, data checks ensured that findings came from the data and not the researchers’ inclinations. In focus groups, reserved participants may be hesitant to contribute to the focus group discussion. To counter this, both moderators were trained to monitor participant contributions to the focus group discussion and to engage all focus group participants in the dialogue. Recruitment of fathers was a challenge because of conflicts with work schedules. In an effort to combat this, we held the father focus group on a weekday evening to increase participation, but few fathers participated. A low male turnout is a common limitation with communities where the male is the primary breadwinner and time off from work often means no pay. Nonetheless, the fathers were articulate and candid.

## Conclusions

Our study addressed research gaps by examining the understudied social and cultural contexts (practices, routines, rules, and beliefs) that shape children’s screen time use among a sample of Latino immigrants living in *colonia* on the U.S.-Mexico border. This sample of parents indicated that *familismo* (family cohesiveness and bonding) and *respeto* influence familial decision-making including screen time practices. *Familismo* and *respeto* are valued constructs in many Latino homes, but most screen time interventions do not include strategies that build and strengthen these values via leisure-time physical activities. Our study findings provide a foundation for further confirmatory research and potential intervention strategies for screen time reduction, specifically, social and cultural dimensions.

## Statement of animal welfare

This article does not contain any studies with animals performed by any of the authors.
